# Dysphagia, Fear of Choking and Preventive Measures in Patients with Huntington's Disease: The Perspectives of Patients and Caregivers in Long-Term Care

**DOI:** 10.1007/s12603-022-1743-6

**Published:** 2022-02-04

**Authors:** K. Kalkers, J.M.G. A. Schols, E.W. van Zwet, R.A.C. Roos

**Affiliations:** 1 Department of Neurology, Leiden University Medical Center, Leiden, The Netherlands; 2 Department of Psychology, Mijzo care organization, location de Kloosterhoeve, Kloosterweg 1, 4941EG, Raamsdonksveer, The Netherlands; 3 Department of Health Services Research, Caphri - Care and Public Health Research Institute, Maastricht University, Maastricht, The Netherlands; 4 Department of Family Medicine, Caphri - Care and Public Health Research Institute, Maastricht University, Maastricht, The Netherlands; 5 Department of Biomedical data sciences, Leiden University Medical Center, Leiden, The Netherlands

**Keywords:** Huntington's disease, dysphagia, fear, cognition, awareness

## Abstract

**Objectives:**

To explore the prevalence of dysphagia and fear of choking in patients with Huntington's disease (HD) as well as preventive measures, both those applied and those not included in managing dysphagia. Also, to investigate related problems encountered by their formal and informal caregivers.

**Design:**

A multi-center observational cross-sectional study

**Setting and Participants:**

158 HD patients, recruited from six Dutch nursing homes specialized in HD, and their formal and informal caregivers

**Measurements:**

Patients were assessed by means of questionnaires enquiring about dysphagia, fear of choking and measures to manage dysphagia. Also, questionnaires were administered about awareness of dysphagia symptoms, cognition and anxiety. Because we expected individuals with greater care dependency to have a higher severity of dysphagia, we distinguished between a care-independent and a care-dependent group of HD patients.

**Results:**

In the total group, 90.5% of HD patients had one or more dysphagia symptoms. The prevalence of FoC in HD patients and the formal and informal caregivers' fears about choking in HD patients was 45.7%, 19.0% and 59.5%, respectively, for care-independent patients and 58.7%, 50.1% and 77.5% for care-dependent patients. The score on the Huntington's Disease Dysphagia Scale was a predictor for fear of FoC in care-independent patients. Speech-language therapy, supervision during eating and drinking and adaptation of food and drink consistency were the most frequently applied measures to manage dysphagia, a combination was used in most HD patients.

**Conclusions:**

In HD patients, the prevalence of dysphagia is high and fear of choking is common among both patients and caregivers. A more severe degree of dysphagia is a predictor of FoC in care-independent HD patients. A combination of measures was used to manage dysphagia in most HD patients.

## Introduction

Huntington's disease (HD) was first described in 1872 by George Huntington (1). This autosomal dominant inherited disease is characterized by unwanted choreatic movements, behavioral and psychiatric disturbances and dementia (2). Prevalence varies widely, the average ranging from 0.42 to 9.71 per 100,000 (3). The mean duration of the disease is 17–20 years and the mean age at onset is 30–50 years with a wide range (2–85) (2). Disease progression results in complete dependency and progressive care needs. At the time of institutionalization, most HD patients are middle-aged (2).

Besides the unwanted choreatic movements, patients often develop hypokinesia, akinesia and rigidity, thereby also affecting the oropharyngeal muscles causing dysphagia (2, 4). Problems with swallowing include lack of coordination, lingual chorea, repetitive swallows, frequent eructations, coughing when eating, and choking on liquids (5). Dysphagia is common in patients with HD, but little is known about its frequency or at what stage it becomes clinically evident (4). The consequences of dysphagia can be serious, including pneumonia, acute respiratory distress and subsequent death (4). Pneumonia is, in fact, the most prominent primary cause of death in HD (6). Dysphagia can, therefore, potentially give rise to fear of choking (FoC) (4, 7), but little is known about this issue in HD (4, 8).

Fear and anxiety are useful emotions which lead to preventive responses (9). Some HD studies have reported reduced fear responses (10, 11). If the patient experiences less fear, this may influence the ability to act safely. In daily practice, we indeed do notice HD patients with dysphagia taking great risks while eating and drinking. Lack of awareness is a possible reason for experiencing less fear, as described by MCusker and Loy: ‘symptoms or the impact are obvious to the objective observer, but the individual does not notice or underestimates them’ (12). As a result of this reduced self-awareness, the person will take more risks (13). In HD, lack of awareness can appear at any stage and in different domains (14). Furthermore, the level of fear that is experienced by HD patients can also be influenced by cognitive factors, such as the ability to evaluate possible consequences of behavior (15), the ability to comprehend information and the awareness of task demands (16).

Little is known about the effect of dysphagia in HD patients on the formal (nurse or physician) or informal (family members or friends) caregivers. Informal caregivers of neurological patients or older people with dysphagia experience a higher anxiety level and increased burden (17, 18). Formal care providers may be prone to overprotection, leading to restrictions which can result in negative consequences, such as decreased hydration and nutrition (19).

As little is known about dysphagia and FoC in HD patients, or about the formal and informal caregivers' fear that the patients might choke, this study aims to explore the prevalence of dysphagia symptoms, the prevalence of FoC in HD patients and their caregivers and the measures currently applied, as well as those not included in managing dysphagia in HD patients. As there is a positive correlation between severity of dysphagia and disease severity (7, 8), we have chosen to focus on HD patients receiving care in nursing homes, i.e. patients within moderate and advanced stages of HD. In addition, because we expect a greater severity of dysphagia in those with higher care dependency, we distinguish between a care-dependent and a care-independent group of HD patients.

The research questions are, therefore:

• What is the prevalence of dysphagia symptoms in care-independent and -dependent HD patients?
• What is the prevalence of FoC in care-independent and -dependent HD patients?
• What is the association between FoC and lack of awareness, cognition and anxiety in care-independent and -dependent HD patients?
• Which measures to manage dysphagia are used and which are missed by care-independent and care-dependent HD patients?
• What is the prevalence of formal and informal caregivers' fear about choking in care-independent and -dependent HD patients?
• Which measures to manage dysphagia are used and which are missing, from the formal and informal caregivers' perspectives?

This study is part of a larger study on FoC and fear of falling in patients with Huntington's disease (20).

## Methods

### Design

This study is an observational, cross-sectional study and part of a larger study on falls and dysphagia carried out in patients with HD, receiving care in one of the Dutch nursing homes specialized in HD, and in their formal and informal caregivers. The extensive protocol of this study has been published elsewhere (20).

### Participants

Patients ≥18 years, with clinically and/or genetically confirmed HD were included. Patients who did not meet the exclusion criteria (20) could not participate. They were recruited from six Dutch HD specialized care organizations; our aim was to reach the maximum number of HD patients. Written informed consent was obtained from all participants or their legal guardian. If patients were unable to answer all questions, only the information provided by their caregivers was used.

One formal and one informal caregiver, closely related to each patient, were asked to participate to assess the prevalence of FoC and define the current and missing measures to prevent choking.

### Assessments

#### Patients

Patient information was derived from the patient, their primary responsible nurse and the speech-language therapist. Patient questionnaires were administered by means of a standardized (semi-) structured interview. In order to standardize the interviews, all questions and answer options were integrated into a standardized flowchart.

In a structured interview with the HD patient, the prevalence of dysphagia symptoms was measured with the Huntington's Disease Dysphagia Scale (HDDS) (21), which contains eleven questions on dysphagia in relation to HD (E.g. 'Do you drool during the day?'). A higher score (with a maximum of 55) represents a more severe degree of dysphagia. FoC was quantified with a single question ('Are you afraid of choking?') requiring a yes-no answer. In addition, the subscale 'fear' of the Swallowing Quality of Life questionnaire (SWAL-QoL-NL) was used (22, 23). The overall scale measures quality of life of patients with oropharyngeal dysphagia, but in the present study, only the subscale fear, which consists of 4 questions, was used. Patients were presented with concerns that people with swallowing problems sometimes mention (E.g. ‘I fear I may start choking when I eat food‘). They indicated on a scale from 1 to 5 how often they experienced each feeling during the last month. In this questionnaire, a higher score reflects less fear. To assess awareness of dysphagia, the method of questionnaire-based discrepancy was used (24). In addition to the HDDS (21) administered to the patient, a separate caregiver's version was administered to a nurse and the discrepancy calculated (patient's score minus caregiver's score). A negative discrepancy score is interpreted as less awareness of dysphagia. A brief cognitive screening tool, the Montreal Cognitive Assessment (25), was used to examine cognition. To measure the level of anxiety a patient is experiencing, the Hospital Anxiety and Depression Scale HADS (26) was used. Currently used measures to manage dysphagia and those that were missed were asked about in an interview. The measures missed were defined as measures that are not applied but that the patient would like to see applied. Answers could be selected from a variety of possible standard measures, but there was also room for alternatives.

In addition, the trained nurse who was primarily responsible completed an online questionnaire about general patient characteristics and dysphagia. In addition to age, gender, height and weight, functional capacity was examined with the Total Functional Capacity Scale, a 5-item clinician rating scale, part of the Unified Huntington Disease Rating Scale (27). The extent to which the patient was care-dependent was examined with the Care Dependency Scale (28), which consists of 16 items based on 15 basic needs and one subjective judgement of care dependency. This scale was used to classify the HD patients as care-independent or care-dependent. A score of 45 or higher means that the patient is classified as care-independent, a score of 44 or lower as care-dependent. Hereafter, these patients will be referred to as independent and dependent. To specify the need for assistance with eating, the item ‘capacity to eat’ of the motor section of the Unified Huntington Disease Rating Scale for advanced patients (UHDRS-FAP) (29) was used. Information about consistency of the food or the use of a PEG tube was registered by the speech-language therapist, using the Functional Oral Intake Scale (FOIS) (30).

#### Informal and Formal caregivers

Subsequently, the prevalence of formal and informal caregivers' fear about choking in HD patients was assessed using a parallel, self-administered caregivers' version of the SWAL-QoL-NL and a single direct question about FoC ('Are you afraid the patient will choke?'). Hereafter, caregivers' fear about choking in HD patients will be referred to as caregivers' FoC. Additionally, questions about measures to manage dysphagia that are currently used and measures missed were asked in parallel with the patients' version.

### Ethical approval

The Medical Ethics Review Committee of VU University Medical Center (2017.445) declared that the Medical Research Involving Human Subjects Act (WMO) does not apply and hence official approval by the committee is not required (31). Written consent was obtained from all participants.

### Statistical analyses

Analyses were performed using the Statistical Package for the Social Sciences (SPSS) version 26. The results are shown for the patient group as a whole and separately for the respective groups of independent and dependent patients, based on the Care Dependency Scale score.

To evaluate the differences between the independent and dependent groups for demographic data and patient characteristics and FoC and SWAL-QoL subscale fear scores, Generalized Linear Models were used. The outcome measures FoC and the SWAL-QoL subscale fear were assessed by the patient themselves, and by their formal and informal caregivers. Because these assessments concern the same patient, they are statistically correlated. To account for this, we used Generalized Estimating Equations (GEE) with an exchangeable correlation structure, to evaluate the differences within the independent and dependent group. Finally, we evaluated the outcome measure FoC in a multivariable logistic regression analysis. Included in the regression model were awareness of dysphagia, cognition, anxiety, age, gender, patients' HDDS, Food consistency and capacity to eat. A p-value ≤0.05 was considered significant.

Multiple imputation for missing data was used. Ten imputations were computed. The imputation model for the HD patients' characteristics and factors associated with FoC included awareness of dysphagia, cognition, anxiety, age, gender, patients' and caregivers' HDDS, capacity to eat, patients' FoC, patients' Swal-Qol-NL, BMI, Care Dependency Scale score and Total Functional Capacity score. For FoC and SWAL-QoL-NL scores, patients' and formal and informal caregivers' FoC, patients' and formal and informal caregivers' Swal-Qol-NL scores, age, gender, food consistency, patients' and caregivers' HDDS, BMI, Care Dependency Scale score and Total Functional Capacity score were included. Finally, in addition to the measures mentioned by patients and formal and informal caregivers, the model for current and missed measures included age, gender, patients' HDDS, Care Dependency Scale score and Total Functional Capacity score.

## Results

### Participants

A total of 245 patients with Huntington's disease meeting the inclusion criteria could be approached for participation, of whom 161 patients, or their legal representatives, gave consent. Three patients died before the start of the assessments, resulting in 158 participants.

General patient characteristics and prevalence of dysphagia symptoms at baseline are shown in table 1. The dependent group had lower functional capacity (p<0.001), a higher need for assistance with eating (<0.001), a higher level of adapted food consistencies (p<0.001) and a lower level of cognitive functioning (p<0.001) compared to the independent group. There was no significant difference between the groups with regard to age (p=0.06), gender (p=0.72), prevalence of dysphagia symptoms (0.43), severity of dysphagia (p=0.16), BMI (p=0.66), anxiety (p=0.60) or awareness of dysphagia symptoms (p=0.07).

**Table 1:** Demographic data and general patient characteristics (means and percentages) of individuals with Huntington's disease

	**All HD patients (n=158)**	**Independent group (n=83)**	**Dependent group (n=75)**	**p-value**
Care-Dependency Scale (SD)	45.2 (17.9)	60.3 (7.4)	28.5 (9.2)	<0.001
Age (SD)	58.8 (12.1)	57.1 (12.8)	60.7 (10.9)	0.06
Male (%)	46.8 *	48.2	45.3 *	0.72
UHDRS Total Functional Capacity (SD)	2.0 (2.5)	3.7 (2.4)	0.2 (0.4)	<0.001
Prevalence of dysphagia (%)	90.5 †	88.7 §§	92.5 †††	0.43
Huntington Disease Dysphagia Scale patient (SD)	18.2 (6.8) †	17.4 (6.3) §§	19.0 (7.2) ‡‡‡	0.16
BMI (SD)	25.6 (15.0) ‡	25.0 (5.3) ∥	26.2 (21.1) §§§	0.66
Capacity to eat (UHDRS-FAP) (%)	§		§	<0.001
0 Normal	35.4	60.2	8.0	
1 Slow but correct	15.3	19.3	10.8	
2 Possible but unable to cut meat	10.2	7.2	13.5	
3 Can eat only with spoon or fingers	10.2	7.2	13.5	
4 Complete help	21.5	1.2	43.9	
5 Gastrostomy but oral food still possible	2.4	0	5.0	
6 Gastrostomy, and oral food impossible	0	0	0	
Functional Oral Intake Scale (%)	∥		∥	<0.001
1 Nothing by mouth	1.3	0	2.7	
2 Tube-dependent with minimal attempts of food or liquid	0.6	0	1.4	
3 Tube-dependent with consistent oral intake of food or liquid	0.6	0	1.4	
4 Total oral diet of a single consistency	18.8	1.3	37.8	
5 Total oral diet with multiple consistencies, but requiring special preparation or compensations	41.6	36.3	47.3	
6 Total oral diet with multiple consistencies without special preparation, but with specific food limitation	9.1	17.5	0	
7 Total oral diet with no restrictions	27.9	45.0	9.5	
MOCA (SD)	12.3 (8.1) **	17.2 (5.6) ***	7.0 (6.9) ∥∥	<0.001
HADS-anxiety (SD)	4.1 (5.3) ††	4.5 (4.8) †††	3 8 (5 9) ****	0.60
Awareness of dysphagia symptoms (SD)	−0.9 (8.1) ‡‡	0.3 (8.2) §§	−2.1 (7.8) ††††	0.07


Scores are based on pooled imputed data; HD=Huntington's Disease; SD=Standard Deviation; UHDRS= Unified Huntington's Disease Rating Scale; BMI=Body Mass Index; UHDRS-FAP = UHDRS for advanced HD patients MOCA=Montreal Cognitive Assessment; HADS=Hospital Anxiety and Depression Scale; Data were unavailable for the following numbers of HD patients: * 1 HD patient; † 60 HD patients; ‡ 12 patients; § 2 patients; ∥ 4 patients; ** 69 patients; †† 65 patients; ‡‡ 59 patients; §§ 13 patients; *** 18 patients; ††† 15 patients; ‡‡‡ 47 patients; §§§ 8 patients; ∥∥ 51 HD patients; **** 50 HD patients; †††† 46 HD patients

### Dysphagia symptoms

In the total group, 90.5% of the HD patients had one or more dysphagia symptoms. In the independent and dependent groups, the prevalence of dysphagia was 88.7% and 92.5%, respectively (p=0.43).

### Fear of Choking

The prevalence of FoC, based on the question ‘are you afraid of choking’, in HD patients was 51.9% (table 2). FoC did not differ significantly between the independent (45.7%) and dependent (58.7%) patients (p=0.53). Patient scores on the SWAL-QoL-NL (17.1 and 13.8, respectively) differed between the two groups (p=0.016), indicating that the independent group experienced less fear. For both the formal and informal caregivers related to the dependent group, a significantly greater amount of fear was found for FoC and the SWAL-QoL-NL, compared to the independent group. Within the independent and dependent groups, FoC and the amount of fear on the SWAL-QoL-NL were significantly higher in the informal caregivers (59.5% and 77.5%, respectively, for FOC and 16.0 and 13.5, respectively, for SWAL-QoL-NL) compared to the formal caregivers (19.0% and 50.1%, respectively, for FOC and 17.8 and 15.9, respectively, for SWAL-QoL-NL).

**Table 2:** Percentages of Fear of choking and total scores of the subscale ‘fear’ of the Swallowing Quality of Life questionnaire in patients with Huntington's Disease and from their formal and informal caregivers' perspective

	**Fear of choking (%)**				**SWAL-QoL-NL fear (SD)**			
	**All HD patients (n=158)**	**Independent group (n=83)**	**Dependent group (n=75)**	**p-value**	**All HD patients (n=158)**	**Independent group (n=83)**	**Dependent group (n=75)**	**p-value**
HD patient	51.9 *	45.7 §	58.7 ††	0.53	15.5 (5.4) *	17.1 (3.7) §	13.8 (6.3) ††	0.016
Formal caregivers	33.8 †	19.0 ∥	50.1 ‡‡	<0.001	16.9 (3.2) §§	17.8 (2.9) ***	15.9 (3.3) ‡‡	<0.001
Informal caregivers	68.0 ‡	59.5 **	77.5 **	0.050	14.8 (4.2) ∥∣	16.0 (3.9) †††	13.5 (4.2) ‡‡‡	0.010
p-value HD patients vs formal caregivers	0.13	0.001	0.69		0.029	0.12	0.09	
p-value HD patients vs informal caregivers	0.18	0.14	0.34		0.35	0.14	0.88	
p-value formal caregivers vs informal caregivers	<0.001	<0.001	0.004		<0.001	0.004	0.001	


Scores are based on pooled imputed data; HD=Huntington's Disease; SWAL-QoL-NL fear=subscale ‘fear’ of the Swallowing Quality of Life questionnaire; SD=Standard Deviation; Data were unavailable for the following numbers of participants * 60 HD patients; † 14 formal caregivers; ‡ 44 informal caregivers; § 13 HD patients; ∥ 10 formal caregivers; ** 22 informal caregivers; †† 47 HD patients; ‡‡ 4 formal caregivers; §§ 13 formal caregivers; ∥∣ 48 informal caregivers; *** 9 formal caregivers; ††† 25 informal caregivers; ‡‡‡ 23 informal caregivers

### Factors associated with Fear of Choking

Patients' HDDS score was found to be a predictor for FoC (p=0.032) in the independent group (table 3). Controlling for awareness of dysphagia, cognition, anxiety, age, gender, FOIS and capacity to eat, showed that independent patients with a higher score on the dysphagia scale were more likely to experience FoC.

**Table 3:** Factors associated with Fear of Choking in independent and dependent patients with Huntington's Disease

**Variable**	**Independent group**			**Dependent group**		
	**O.R.**	**95% C.I.**	**p-value**	**O.R.**	**95% C.I.**	**p-value**
Awareness of dysphagia	0.94	0.81–1.09	0.39	0.91	0.70–1.20	0.49
MOCA	1.01	0.87–1.17	0.93	1.07	0.82–1.41	0.59
HADS-anxiety	1.17	0.98–1.40	0.08	1.01	0.76–1.34	0.93
Age	0.99	0.95–1.05	0.82	1.03	0.94–1.13	0.51
Gender	3.67	0.90–14.96	0.07	1.41	0.19–10.38	0.73
HDDS	1.25	1.02–1.54	0.032	1.31	0.87–1.97	0.19
FOIS	0.84	0.31–2.29	0.73	0.96	0.37–2.50	0.93
Capacity to eat (UHDRS-FAP)	1.39	0.46–4.17	0.55	1.05	0.50–2.20	0.90


Scores are based on pooled imputed data; O.R.= Odds Ratios; C.I.=Confidence Interval; MOCA= Montreal Cognitive Assessment; HADS-anxiety= Hospital Anxiety and Depression Scale part Anxiety

### Measures to manage dysphagia

The average number of measures taken to manage dysphagia, according to HD patients, was 3.4 in the independent group and 5.3 in the dependent group (p=0.004). According to the formal caregivers in the independent group, an average of 3.5 measures was used, in contrast to 4.8 measures in the dependent group (p=0.006). These numbers were 3.5 and 4.9, respectively, for informal caregivers (p=0.001).

In the independent group, speech-language therapy was the most frequently used measure to manage dysphagia, according to the HD patients (52.2%) and the formal caregivers (53.1%). For the informal caregivers, this was supervision during eating and drinking (50.1%). In the dependent group, the patients mentioned that speech-language therapy was most frequently used (62.7%), while according to the formal (66.3%) and informal (65.3%) caregivers, this involved adapted consistencies of food and drinks. In addition to a list of possible standard measures, participants could also name other alternative measures currently applied. These measures were mentioned by HD patients: securing the plate to the table (0.6%), concentrating while eating (0.6%), flushing food down by drinking (0.6%), taking medicines with custard (0.6%). Risk acceptance (1.8%), eating at a different time (0.6), cutting the food into small pieces (0.6%) and tube-feeding (0.6%) were mentioned by formal caregivers and adjusting eating posture (1.2%), cutting the food into small pieces (0.6%, further adjustment of consistencies of food and drinks (0.6%) and tube-feeding (0.6%) were mentioned by informal caregivers as alternative measures. Then participants were asked what measure was not applied currently, but they would like to see applied. HD patients in the independent group most frequently indicated that patient education (16.7%) should be applied more often to manage dysphagia. ‘Agree with resident and/ or relatives about preventive measures’ was most frequently (11.9%) missed by formal caregivers and ‘Use of an assistive device when eating or drinking’ (24.6%) by informal caregivers. HD patients in the dependent group reported ‘Patient education’ ((58.1%) as the measure most frequently missed. The formal caregivers quoted ‘adapted consistencies of food and drinks’ (7.2%) and the informal caregivers ‘use of an assistive device when eating or drinking’ (26.4%). In addition to standard measures, no alternative missing measures were mentioned by HD patients and formal caregivers. Informal caregivers indicated that they missed evaluation by a dietitian and that speech-language therapy is discontinued too soon.

## Discussion

The approximately 90% prevalence of dysphagia found in this study is higher than in previous studies with HD patients, which found rates of between 35% and 80% (5, 32, 33). Because of different study methods, such as inclusion criteria and diagnostics, it is difficult to compare these findings with the results of our study. Although we expected the dependent group to have a higher prevalence and greater severity of dysphagia symptoms than the independent group, no significant differences were found between the groups. A greater need for assistance with eating and a higher level of adapted food consistencies in the dependent group compared to the independent group did, however, indicate a greater degree of swallowing problems in the dependent group. A possible explanation for this discrepancy is the lower level of cognitive functioning in the dependent group, resulting in less reliable completion of the questionnaire on swallowing difficulties.

**Table 4:** Percentages of current and missing measures to manage dysphagia from the perspective of patients with Huntington's Disease and their formal and informal caregivers

**Measures**	**Independent group (n=83)**						**Dependent group (n=75)**					
	**Current measures in %**			**Missing measures in %**			**Current measures in %**			**Missing measures in %**		
	**HD Patients †**	**Formal Caregivers ‡**	**Informal Caregivers §**	**HD Patients †**	**Formal Caregivers ‡**	**Informal Caregivers §**	**HD Patients ∥**	Formal Caregivers **	**Informal Caregivers ††**	**HD Patients** ∥	Formal Caregivers **	**Informal Caregivers ††**
Evaluation of current medication	14.0	16.9	23.3	0	8.3	18.1	36.7	20.1	24.8	0	3.1	14.8
Involvement speech language therapist	52.2	53.1	47.5	9.8	7.3	22.3	62.7	55.3	45.1	34.3	5.1	23.1
Supervision when eating and drinking	48.7	52.3	50.1	0	6.0	16.5	52.0	65.3	64.9	0	3.2	17.9
Support with eating or drinking (such as cutting up food)	34.7	25.9	35.4	0	10.6	17.8	55.6	46.9	53.6	0	5.9	20.3
Complete assistance with eating or drinking	8.8	6.3	16.5	0	11.0	15.9	34.9	47.3	49.6	0	5.7	17.9
Use of an assistive device when eating or drinking	23.4	20.7	25.7	12.2	11.3	24.6	55.3	40.5	41.2	36.3	6.4	26.4
Alarm	27.2	16.6	28.3	10.6	0	0	46.1	8.0	30.3	39.6	0	0
Patient education	51.1	43.7	38.3	16.7	4.6	12.6	48.5	39.3	40.7	58.1	3.3	8.1
Adapted consistencies of food and drinks	34.7	28.7	34.1	0	6.3	16.3	52.7	66.3	65.3	0	7.2	16.8
Agree with resident and/or relatives about preventive interventions	17.7	25.5	20.0	0	11.9	22.9	45.7	27.9	32.0	0	4.4	24.9
Safety adaptation of environment	27.8	37.3	20.0	0	10.7	18.4	38.3	46.4	33.9	0	5.5	14.0
Use of restraints	0	9.2	11.7	0	0	18.2	0	4.7	12.5	0	0	18.3
Other interventions	13.0	10.8	16.5	14.0	6.8	19.8	28.1	9.9	22.9	46.5	2.9	18.4
Patient refuses all interventions	0	7.3	18.1	n.a.	n.a.	n.a.	0	2.9	15.9	n.a.	n.a.	n.a.
No interventions	20.6	25.9	28.4	81.4	80.0	70.7	36.5	9.1	16.5	61.7	87.1	65.9


Percentages are based on pooled imputed data; *HD=Huntington's Disease; Data were unavailable for the following numbers of participants: † 14 HD patients; ‡ 10 formal caregivers; § 24 informal caregivers; ∥ 47 HD patients; ** 4 formal caregivers; †† 20 informal caregivers;

FoC, based on the question ‘are you afraid of choking’, was reported by approximately 50% of the HD patients in this study and again there were no significant differences between the groups. However, when asked about specific concerns, such as choking on food or beverages or developing pneumonia, with the SWAL-QoL-NL, patients in the dependent group were significantly more concerned. Although dysphagia in HD is believed to be related to anxiety (4, 7), as yet, little is known in literature about FoC in HD. FoC may have been triggered before the patient developed significant dysphagia symptoms himself, by witnessing relatives (4) or other patients in the nursing home with swallowing problems.

Within the independent and dependent groups, most FoC was found in the informal caregivers and less in formal caregivers; between the groups, most FoC is reported among the caregivers of the dependent group. Although there are no studies describing the prevalence of formal and informal caregivers' fear of choking in HD, several studies have found higher levels of burden and anxiety in informal caregivers related to patients with conditions such as dementia, MS, stroke, and dysphagia (17, 18). Reasons cited include feelings of incompetence and the balance between safety concerns on the one hand and patients' food preferences and nutritional status on the other. Specific reasons for anxiety in HD may include actual experiences with dysphagia in relatives and the progressive nature of the disease.

This study found that patients' HDDS score is a predictor of FoC in care-independent HD patients. Similar results were found in a study where the SWAL-QoL fear of eating score and the swallowing disturbance questionnaire score correlated (8). This again emphasizes the relationship between dysphagia and psychological burden, such as fear (34). A study of nursing home and hospital patients with dysphagia showed that in a large proportion of the patients, the quality of life was negatively affected by psychological and social consequences of dysphagia (34).

We found no association between FoC and lack of awareness, cognition and anxiety. Perhaps this relationship could be found with a validated questionnaire to measure awareness of swallowing risks, analogous to the Self-awareness of Fall Risk Questionnaires (35), or the use of specific cognitive domains, such as memory or executive function; therefore, this requires more attention in future studies.

‘Involvement of speech-language therapist’, ‘Supervision when eating or drinking’ and ‘Adapted consistencies of food and drinks’ were the measures most frequently used to manage dysphagia in this study; in most patients, a combination was applied. On the basis of a review of the scarce literature about management of dysphagia in HD, compensation strategies are recommended; also, pharmacological and rehabilitation treatments should be further investigated (36). No information was found about treatments to cope with FoC.

The strength of this study is the fact that so many participants with this rare disorder (3) could be included. However, we encountered some challenges as we selected patients with moderate and advanced stages of HD. As cognitive performance deteriorates during the disease (37), we realized that not all patients were able to fill in their questionnaire by themselves. Although questioning cognitively impaired patients is often avoided, our goal was to have as many patients as possible answer the questions in a valid manner. Therefore, we used standardized interview-based questionnaires instead of self-completion questionnaires, because of evidence of advantages in terms of validity, reliability and completion percentages in persons with cognitive impairments (38). Using this strategy, as expected, many values were still missing, particularly for care-dependent HD patients. Therefore, multiple imputation for the missing data was used. Consequently, the results of this group were less reliable, being more of an indication than a hard outcome. However, data provided by the formal and informal caregivers of these patients supplemented information about FoC in HD. Thus, although results about FoC from care-dependent patients should be interpreted with caution, this study provides initial insight into an important area of concern for this population which is often excluded from studies.

## Conclusions

In HD patients, the prevalence of dysphagia is very high and FoC is common among patients and their formal and informal caregivers. A more severe degree of dysphagia is a predictor of FoC in care-independent HD patients. To manage dysphagia, a combination of measures was used in most HD patients.
